# Increasing the Dose of Onabotulinum Toxin A From 400 to 600 Units for Post-stroke Spasticity: A Retrospective Study on Safety and Muscle Selection

**DOI:** 10.7759/cureus.96703

**Published:** 2025-11-12

**Authors:** Gentaro Hashimoto, Masahiro Abo, Shin Suzuki, Kaho Tamura, Kentaro Yoshida

**Affiliations:** 1 Rehabilitation Medicine, The Jikei University School of Medicine, Tokyo, JPN

**Keywords:** fugl-meyer assessment-upper extremity, onabotulinum toxin a injections, physical medicine and rehabilitation, post-stroke recovery, quality of life(qol)

## Abstract

In Japan, the approved maximum dosage of Onabotulinum Toxin A (BoNT-A) (BOTOX, Allergan plc, Irvine, CA, USA) for upper and lower limb spasticity was increased to 600 units in November 2022. As reports on this high dosage are rare internationally, this study aimed to confirm its safety and evaluate the muscles administered. The study included 24 stroke patients (18 men, six women) treated with 600 units of BoNT-A between December 2024 and January 2025. Medical records were reviewed for treatment history, including the number of injection cycles and target muscles. On the final injection day, patients were verbally surveyed about side effects of the dose increase (from 400 to 600 units), changes in spasticity, functional improvement, and their intent to continue the regimen. The patients had been receiving BoNT-A therapy for a mean duration of 7.5 years. Eight key muscles, including the pectoralis major, biceps brachii, hamstrings, and gastrocnemius, were injected in over 70% of cases. The study found no side effects or adverse events resulting from the dose increase in any patient. Furthermore, all 24 patients reported a greater reduction in spasticity and noted that it was easier to use their paralyzed side. All patients expressed their intention to continue receiving the 600-unit dose. In conclusion, given the long-term treatment history, lack of adverse events, and unanimous patient-reported benefits, the safety of increasing the BoNT-A dose to 600 units for limb spasticity is considered to be assured.

## Introduction

In Japan, Onabotulinum Toxin A (BoNT-A) (BOTOX®, Allergan plc, Irvine, CA, USA) was approved for insurance coverage for upper and lower limb spasticity at the end of October 2010, marking 15 years of its use. Initially, international reports on BoNT-A therapy for upper limb spasticity primarily focused on the (i) reduction of spasticity (improvement in Modified Ashworth Scale scores) and (ii) improvement in passive range of motion, with its efficacy established through numerous double-blind controlled trials. Meanwhile, although some reports described improvements in activities of daily living (ADL), evidence regarding improvements in active upper limb motor function was limited to a few case reports.

Against this backdrop, and prior to post-marketing surveillance in Japan, we published in English that the combination of BoNT-A therapy and self-administered training promotes central functional recovery even in cases of severe paralysis [1,2]. Since then, numerous studies have been published verifying the efficacy of combining BoNT-A therapy with physical therapy [3,4], and it is now widely recognized that this combination contributes to functional improvement. The Japanese Stroke Treatment Guidelines 2021 also recommend BoNT-A therapy for reducing post-stroke upper and lower limb spasticity and for improving motor function [5].

Regarding dosage, the maximum approved dose of BoNT-A for upper limb spasticity in Japan was 240 units for a long time since its approval in 2010, but it was increased to 400 units in December 2019 [6]. Furthermore, the maximum dose for upper and lower limb spasticity was raised from 360 units to 600 units in November 2022 [7]. As the maximum approved dose in many other countries is 400 units, clinical reports on the use of 600 units were previously considered scarce [7]. However, several retrospective studies have reported on the safety and efficacy of high-dose (≥600 U) administration, suggesting its feasibility in specific patient groups [8,9]. To our knowledge, there are only a few reports that specifically analyze the background and breakdown of injected muscles in patients whose dosage was escalated from 400 to 600 units to examine real-world clinical trends. Therefore, this study aimed to confirm the safety of administering 600 units of BoNT-A in a real-world clinical setting and to clarify the actual practices of muscle selection during this specific dose escalation.

## Materials and methods

At our institution, we have been using BoNT-A for upper and lower limb spasticity since the end of October 2010. Currently, we administer BoNT-A for upper and lower limb spasticity to approximately 800 patients per year. The change in the maximum dosage from 400 units to 600 units was implemented in November 2022.

Participants

The subjects were 24 patients (18 men, six women) who received an increased dose of BoNT-A from 400 to 600 units for post-stroke spasticity at our outpatient rehabilitation clinic between November 2022 and January 2025. All patients were independently ambulatory indoors using a cane and a lower limb orthosis, and their ADL were at an independent level. This study analyzed all consecutive patients who met these criteria during the study period. No patients were excluded due to severe cognitive impairment, comorbidities, or discontinuation of BoNT-A during the observation period.

Study design and data collection

This study is a retrospective case series based on a review of medical records. The following information was collected from the medical records of the 24 target patients: date of onset of the primary disease (stroke), start date of BoNT-A therapy, start date and number of sessions of 400-unit administration, and start date and number of sessions of 600-unit administration during this study period.

Evaluation items

Transition of Injected Muscles and Dosages

We investigated the following at the time of the first and last 400-unit injections, as well as the first and last 600-unit injections during the study period, to examine trends: (i) types of muscles injected, (ii) injection frequency for each muscle (as a percentage of all patients), and (iii) the distribution rate of the dose for each muscle relative to the total dose (%).

Safety and Subjective Evaluation

The presence of adverse events (including systemic events such as dysphagia or generalized fatigue, as well as local events) associated with the dose increase to 600 units was confirmed through routine verbal questioning at each visit and a review of medical records. Additionally, on the day of the final injection during the study period, changes in spasticity, a sense of improvement in motor function, and the desire to continue with 600-unit injections were confirmed through verbal questioning.

Upper Limb Motor Function

To assess the severity of upper limb motor paralysis, Fugl-Meyer Assessment (FMA, upper limb section, maximum score of 66) [10] scores before the start of BoNT-A therapy and at the final time point of the 600-unit administration period were compared. Furthermore, long-term changes in functional level were evaluated using the five-level severity classification by Hoonhorst et al. [11] (no upper-limb capacity: FMA ≤22, poor capacity: 23-31, limited capacity: 32-41, notable capacity: 42-52, full upper-limb capacity: ≥53).

Statistical analysis

Changes in FMA scores (pre-BoNT-A therapy vs. final 600-unit time point) were compared using the Wilcoxon signed-rank test, as the data were not normally distributed. Statistical significance was set at p<0.05. All analyses were performed using EXCEL Toukei Ver. 8.0 (Social Survey Research Information Co., Ltd., Tokyo, Japan).

Ethical considerations

This study was conducted after obtaining written consent from the participating patients and receiving approval from the ethics committee of The Jikei University School of Medicine (Approval No. 26-377(7883)).

## Results

Patient characteristics

The characteristics of the 24 patients (18 men, six women) are shown in Table 1. The mean age at stroke onset was 45.5±10.3 years. The mean time from onset to the first BoNT-A injection was 3.8 years, to the start of 400-unit injections was 8.2 years, and to the start of 600-unit injections was 11.3 years. The 400-unit dose was administered an average of 10.4 times, and the 600-unit dose was administered an average of 7.2 times.

**Table 1:** Descriptive Statistics of all Patients at Pretreatment BoNT-A: Onabotulinum Toxin A; FMA: Fugl-Meyer assessment. n=24. Data are presented as mean±standard deviation for continuous variables and n (%) for categorical variables.

Variables	N (%) / Mean±SD
Patient Characteristics	
Age at survey, years	58.3±9.9
Age at onset, years	45.5±10.3
Sex, n (%)	
Female	8 (33.3)
Male	16 (66.7)
Type, n (%)	
Cerebral Infarction	8 (33.3)
Intracerebral Hemorrhage	16 (66.7)
Paralyzed side, n (%)	
Left	6 (25.0)
Right	18 (75.0)
FMA Score	
No upper-limb capacity	15 (62.5)
Poor capacity	3 (12.5)
Limited capacity	4 (16.7)
Notable capacity	1 (4.2)
Full upper-limb capacity	1 (4.2)
Duration from Onset, months	
To first consultation	35.8±35.2
To initiation of BoNT-A	45.3±52.1
To start of 400-unit dose	97.4±53.1
To start of 600-unit treatment	135.5±64.6
Number of Injections	
400-unit doses	10.4±4.4
600-unit treatments	7.2±1.7

Based on the FMA scores before the start of BoNT-A therapy, the severity classification of upper limb paralysis was "no upper-limb capacity" (FMA≤22) in 15 patients (62%), which was the most common, followed by "limited capacity" (32-41 points) in four patients (17%), and "poor capacity" (23-31 points) in three patients (13%), indicating that a majority of cases had severe paralysis (Table 1).

Transition of injected muscles and dosages

A total of 20 different muscles were identified as injection targets. Among them, eight muscles (pectoralis major, latissimus dorsi, biceps brachii, flexor carpi radialis, flexor digitorum superficialis, hamstrings, soleus, and gastrocnemius) were consistently targeted in over 70% of patients throughout both the 400-unit and 600-unit administration periods (Table 2). These eight major muscles tended to receive a relatively high dose of 40 units or more (Table 3).

**Table 2:** Percentage of 400 or 600 Unit Injections into Each Muscle in 24 Subjects

Muscle for injection	Initial 400 units	Final 400 units	Initial 600 units	Final 600 units
Pectoralis major	58.3	83.3	91.7	95.8
Latissimus dorsi	41.7	41.7	70.8	83.3
Teres major	8.3	0.0	8.3	16.7
Biceps brachii	83.3	87.5	79.2	70.8
Triceps brachii	4.2	4.2	54.2	54.2
Brachialis	25.0	25.0	16.7	58.3
Flexor carpi radialis	79.2	87.5	95.8	87.5
Flexor carpi ulnaris	33.3	12.5	25.0	16.7
Flexor digitorum superficialis	75.0	62.5	75.0	62.5
Flexor digitorum profundus	16.7	8.3	12.5	8.3
Brachioradialis	0.0	4.2	8.3	4.2
Palmaris longus	8.3	0.0	4.2	0.0
Adductor pollicis	29.2	37.5	62.5	54.2
Flexor pollicis longus	20.8	12.5	25.0	16.7
Pronator teres	25.0	20.8	25.0	16.7
Hamstring	50.0	70.8	87.5	79.2
Soleus	70.8	70.8	100.0	100.0
Gastrocnemius	58.3	58.3	87.5	100.0
Flexor digitorum brevis	8.0	4.0	4.2	8.3
Flexor digitorum longus	4.0	0.0	4.2	4.2
Data are percentage of patients in whom BoNT-A was injected in the indicated muscle				

**Table 3:** Percentage of Injection Volume per Muscle for 400 or 600 Units in 24 Subjects

Muscle for injection	Initial 400 units	Final 400 units	Initial 600 units	Final 600 units
Pectoralis major	7.8	11.2	9.0	10.8
Latissimus dorsi	5.2	5.2	6.6	6.9
Teres major	1.0	0.0	0.7	1.4
Biceps brachii	13.0	12.5	8.0	6.3
Triceps brachii	0.5	0.5	4.5	4.5
Brachialis	3.1	3.1	1.4	4.9
Flexor carpi radialis	9.9	10.7	7.8	7.3
Flexor carpi ulnaris	4.2	1.6	2.1	1.4
Flexor digitorum superficialis	11.5	8.3	6.9	6.6
Flexor digitorum profundus	2.1	1.0	1.0	0.7
Brachioradialis	0.0	0.5	0.7	0.3
Palmaris longus	1.0	0.0	0.3	0.0
Adductor pollicis	3.6	3.9	5.0	4.3
Flexor pollicis longus	2.6	1.3	2.1	1.2
Pronator teres	3.1	2.3	2.1	1.4
Hamstring	8.3	15.1	15.6	13.5
Soleus	9.9	12.0	12.7	13.0
Gastrocnemius	11.5	10.4	12.7	14.4
Flexor digitorum brevis	1.0	0.3	0.3	0.7
Flexor digitorum longus	0.5	0.0	0.3	0.3
Data are percentage of patients in whom BoNT-A total volume was injected in the indicated muscle				

Safety and subjective evaluation

In all 24 cases, no adverse events (local or systemic) were observed following the dose increase from 400 to 600 units. The mean duration of BoNT-A therapy was long, at 7.5±3.6 years (range: 0.67-11.3 years). Subjective feedback regarding the dose increase was positive; all patients reported a further reduction in spasticity and improved ease of use of the paretic side with the increased dose, and all wished to continue receiving regular 600-unit injections in the future.

Changes in upper limb motor function

Compared to pre-BoNT-A therapy (median FMA score: 22, interquartile range (IQR): 10-33), the FMA scores at the final time point of the study period (median FMA score: 26, IQR: 14-38) showed a statistically significant improvement (Wilcoxon signed-rank test, p=0.002). All patients' scores improved or were maintained; no cases of deterioration were observed. According to the severity classification by Hoonhorst et al. [11], a total of six patients (25%) improved by one severity level: three out of the 15 patients with "no upper-limb capacity" improved to "poor capacity," one out of three patients with "poor capacity" improved to "limited capacity," and two out of four patients with "limited capacity" improved to "notable capacity." The remaining 18 patients (75%) showed improvement in FMA scores but did not cross a severity classification threshold. Particularly in cases with higher severity, functional improvement was limited, and treatment was often continued with the primary goal of maintaining ADL.

## Discussion

In December 2019, the usage limit of BoNT-A in Japan was increased to 400 units. While the maximum dose of BoNT-A available for treating upper limb spasticity is 400 units in many countries, few studies have demonstrated the optimal use of this dosage. We previously showed that 400-unit injections of BoNT-A can alleviate muscle tone across a wide range of sites and improve functional impairment [6]. Although the efficacy of the 600-unit dose used in this study has been confirmed in clinical trials [7] and other retrospective studies, there are no studies in the literature that present the circumstances of patients who increased their dose from 400 to 600 units and examine the associated trends. Therefore, we believe this case series report is valuable. In our study, no patient experienced side effects from increasing the dose from 400 to 600 units. All patients reported a subjective sense of greater reduction in spasticity and found it easier to use their paretic side. Thus, this investigation alone has observed that BoNT-A has been administered over a long period, averaging 7.5±3.6 years, and has confirmed the intention to continue with regular 600-unit doses in the future. Although the safety of 600 units has been reported in a clinical trial by Francisco et al. [7], we believe this report clinically substantiates the safety of the dose increase.

We utilize BoNT-A extensively and have identified four crucial components in our strategy for post-stroke sequelae treatment: (i) sharing realistic treatment goals, (ii) combining with rehabilitation therapy, (iii) treating with a sufficient dosage, and (iv) providing continuous botulinum toxin treatment. Among them, we believe that sharing realistic treatment goals with the patient is the most important. That is, it is crucial to consult with the patient to decide whether the goal of BoNT-A treatment is to reduce care burden and improve quality of life (QOL) or to aim for functional improvement [1-4,6].

We investigated the relationship between upper limb motor function recovery and subjective physical symptoms in 47 patients who had received BoNT-A treatment and occupational therapy more than 20 times on an outpatient basis after stroke onset [11]. Subjective physical state was analyzed using a Visual Analog Scale score on an eight-item questionnaire. Recovery of upper limb motor function was detected by calculating the change (delta) in the FMA score, and an ordered logistic model analysis was used to examine the association between the delta FMA score and the subjective agreement with each item. This study suggested that patients who received long-term BoNT-A treatment and occupational therapy experienced enhanced pain relief in the upper limbs and reduced insomnia after injection, regardless of motor function recovery. This indicates that patients with severe paralysis often use the treatment with the goal of improving care burden and QOL [12]. In the current study as well, while improvements in QOL and functional maintenance were clearly observed with the dose increase, a significant change in the disability severity classification occurred in only 25% of patients. This can be attributed to the high prevalence of severe paralysis (62% with "no upper-limb capacity" in the five-level classification of pre-BoNT-A upper limb function) and, as shown in our previous report [12], reflects the different goals desired by patients, with a longer injection period for QOL purposes in more severe cases rather than for functional improvement. An exploratory study by Lagalla et al. [4] of 28 stroke patients treated for over two years and observed for three years to evaluate the effects of BoNT-A on moderate or severe upper limb spasticity yielded similar results; while ADL improved in all subjects, motor dexterity scores improved in only eight out of 28 patients. Nevertheless, it is considered that patients are accepting of their individual levels of improvement, and we judge that there is a desire to continue regular BoNT-A treatment over the long term. This supports the importance of an appropriate treatment strategy according to severity, as advocated by Woodbury et al. [3]. An interesting finding from our muscle selection data (Table 2) was the increased injection rate for the triceps brachii, while the rate for its antagonist, the biceps brachii, remained stable. This may suggest that as the primary flexor spasticity (biceps) is managed, antagonist spasticity (triceps) or co-contraction around the elbow becomes a more apparent or functionally limiting target, necessitating treatment to improve active control or ease of use.

The "no upper-limb capacity" group is classified as having an FMA score of 22 or less. These patients often face difficulties when the goal is improvement [3,12]. Patients in this group have severe upper limb paralysis, and spasticity and contracture often coexist, making improvement a lengthy process. A proactive attitude is essential, but the training typically provided a few times a week at day care centers in Japan is insufficient in volume. It is necessary to incorporate creative rehabilitation exercises into daily life. Woodbury et al. [3] also state the importance of thoroughly assessing the upper limb paralysis of 512 post-stroke patients and providing training appropriate for their severity level. Continuous and correct rehabilitation therapy combined with BoNT-A therapy is also necessary. In most cases of severe spastic paralysis, improvement starts from the shoulder, i.e., proximally. Therefore, if spasticity is strong in the proximal areas, we focus BoNT-A injections there, and the basics of rehabilitation training involve instructing patients on correct and thorough stretching and exercises to improve shoulder flexion function [1,2,12]. This explains why a large amount of BoNT-A is needed in the proximal part, and we believe it was deemed necessary for treatment improvement to increase the upper limb dose from 240 to 400 units and the combined upper and lower limb dose from 360 to 600 units. A key clinical implication of the 600-unit dose is the ability to concurrently treat these functionally crucial proximal muscles (e.g., pectoralis major, latissimus dorsi) without compromising the dosage required for distal upper limb or lower limb muscles, which was a limitation of the 400-unit ceiling. Thus, it is understandable that, as shown in this study, injections are frequently administered to the pectoralis major and latissimus dorsi. It is also noteworthy that the tibialis posterior (TP), a common target for lower limb spasticity, was not among the frequently injected muscles in this cohort. This may be attributed to the functional status of our participants (all ambulatory indoors), where spasticity in the gastrocnemius and soleus was prioritized as the primary contributor to gait disturbance (e.g., equinus foot) over pes varus deformity, which is often targeted via TP injection.

The limitations of this study include the small number of cases (n=24), its retrospective nature, and the absence of a control group. Further limitations include: (1) the retrospective, open-label design, where evaluators (clinicians) were not blinded to the treatment; (2) the reliance on subjective verbal reports for outcomes like "spasticity reduction" and "ease of use," without standardized scales (e.g., Modified Ashworth Scale, Visual Analog Scale); (3) the lack of a detailed analysis on the precise distribution of the additional 200 units (e.g., upper vs. lower limb, proximal vs. distal) during dose escalation. Although we believe the long-term data spanning over 10 years has clinical value, the results of this study should be considered preliminary. In the future, it will be necessary to accumulate more cases and conduct detailed investigations with a prospective, controlled, and blinded study design, incorporating standardized functional and subjective outcome measures. BoNT-A has been approved for use at 600 units under insurance coverage in Japan since November 2022. Its use became possible in Japan after its safety was confirmed in the Adult Spasticity International Registry (ASPIRE) Study [7], and to date, it has been widely used at our facility and across Japan without reports of problematic side effects. While it may seem somewhat unusual to report on this, we believe this study is valuable as it reconfirms safety with consolidated clinical data and reports on changes in the injected muscles, for which there are no previous reports.

Furthermore, to enhance the effects of BoNT-A therapy, combination with not only rehabilitation but also with extracorporeal shockwave therapy, repetitive peripheral magnetic stimulation, and repetitive transcranial magnetic stimulation (rTMS) is reported to be effective [13]. rTMS was administered to 1,716 post-stroke patients (876 cerebral infarction (CI) patients, 840 intracerebral hemorrhage (ICH) patients) and was shown to be effective in improving manual dexterity by reducing spasticity and promoting isolated movements for moderate to mild upper limb dysfunction in the chronic phase, regardless of whether the cause was cerebral infarction or cerebral hemorrhage. However, it did not show significant effects in severe paralysis. Therefore, BoNT-A therapy can be used to reduce severe spasticity, and further improvement in upper limb function can be expected with the addition of rTMS. The effects of these combined therapies must also be thoroughly evaluated in the future. In conclusion, based on this retrospective case series, increasing the BoNT-A dose to 600 units for post-stroke spasticity appears to be a safe and well-tolerated treatment option that contributes to improved QOL and functional maintenance. When increasing the dose, it is crucial to clarify individual patient treatment goals and select appropriate muscles, including proximal ones.

## Conclusions

In this study, no side effects were observed in any case on increasing the dose from 400 to 600 units. All patients reported feeling a greater reduction in spasticity with the increased dose and noted that it was easier to use their paralyzed side. Although no side effects from the dose increase were observed in this study, it is necessary to accumulate more cases for more detailed investigations in the future. When performing BoNT-A therapy, it is crucial to clearly determine whether the goal is functional improvement or QOL enhancement and to further consider which muscles should be injected with what dosage.
